# Effects of Inoculation With *Acinetobacter* on Fermentation of Cigar Tobacco Leaves

**DOI:** 10.3389/fmicb.2022.911791

**Published:** 2022-06-17

**Authors:** Tianfei Zheng, Qianying Zhang, Qiaoyin Wu, Dongliang Li, Xinying Wu, Pinhe Li, Quanwei Zhou, Wen Cai, Juan Zhang, Guocheng Du

**Affiliations:** ^1^School of Biotechnology, Jiangnan University, Wuxi, China; ^2^Key Laboratory of Industrial Biotechnology, Ministry of Education, School of Biotechnology, Jiangnan University, Wuxi, China; ^3^Science Center for Future Foods, Jiangnan University, Wuxi, China; ^4^Cigar Fermentation Technology Key Laboratory of China Tobacco, China Tobacco Sichuan Industrial Co., Ltd., Chengdu, China

**Keywords:** cigar tobacco leaves, inoculation, microbial community, flavor, *Acinetobacter*

## Abstract

Metabolic activity of the microbial community greatly affects the quality of cigar tobacco leaves (CTLs). To improve the quality of CTLs, two extrinsic microbes (*Acinetobacter* sp. 1H8 and *Acinetobacter indicus* 3B2) were inoculated into CTLs. The quality of CTLs were significantly improved after fermentation. The content of solanone, 6-methyl-5-hepten-2-one, benzeneacetic acid, ethyl ester, cyclohexanone, octanal, acetophenone, and 3,5,5-trimethyl-2-cyclohexen-1-one were significantly increased after inoculated *Acinetobacter* sp. 1H8. The inoculation of *Acinetobacter* sp. 1H8 enhanced the normal evolutionary trend of bacterial community. The content of trimethyl-pyrazine, 2,6-dimethyl-pyrazine, and megastigmatrienone were significantly increased after inoculated *Acinetobacter indicus* 3B2. The inoculation of *Acinetobacter indicus* 3B2 completely changed the original bacterial community. Network analysis revealed that *Acinetobacter* was negatively correlated with *Aquabacterium*, positively correlated with *Bacillus*, and had significant correlations with many volatile flavor compounds. This work may be helpful for improving fermentation product quality by regulating microbial community, and gain insight into the microbial ecosystem.

## Introduction

Traditional fermentation processes, such as Chinese liquor, cigar, and fermented vegetable, are mainly driven by complex microbial communities (Song et al., 2017; Liu et al., 2019; Smyth et al., 2019). However, these processes are long and uncontrolled (Jin et al., 2017; Wu et al., 2021). Modern artificial fermentation has been trying to control these processes by adjusting the fermentation conditions or adding specially formulated starters to the natural fermentation system (Shilei et al., 2019). These methods were widely used in the traditional multi-species fermentation industry because of their various advantages, including shortening fermentation times, improving product qualities, and improving safety. For example, the pH condition considerably affected hydrogen fermentation, hydrogen gas was efficiently produced with unconditioned anaerobic sludge when the pH was adjusted to 6.0 throughout the culture period (Kawagoshi et al., 2005). When *Debaryomyces hansenii* and *Yarrowia lipolytica* were incorporated into the cheese as part of the starter, the cheese could significantly shorten the ripening time while maintaining a good strong flavor (Ferreira and Viljoen, 2003). The inoculation of *Bacillus licheniformis* could affect the microbial community structure and enzyme activity of Daqu, and increased the content of pyrazines and aromatic compounds (Wang et al., 2017). Additionally, the inoculation of *Saccharomyces uvarum* and *Saccharomyces servazzii* significantly improved the quality of Chinese liquor (Wu et al., 2016). However, adjusting fermentation conditions is sometimes not effective, because some traditional fermentation processes have been adjusted thousands of times in their long history. Exogenous addition of microorganisms does not always produce positive results, because inoculated microorganisms cannot promote the evolution of the community towards favorable product fermentation (Wu et al., 2016).

The main reason for the failure of externally added microbes is that microbial interactions between the inoculated microbes (extrinsic) and the native microbes (intrinsic) lead to incomplete structure and function of microbial communities. Microbial interactions, such as symbiotic, synergistic, predation, parasitic, and competitive, can greatly affect the metabolic activity of the microbial communities (Ghosh et al., 2016; Nawaz et al., 2022; Pierce and Dutton, 2022). Rational addition of exogenous microorganisms to activate interactions between microorganisms could strengthen the metabolic capacity of the microbial community. However, it is difficult to determine whether the addition of microbes can promote the fermentation of product unless the microbial interaction is deeply studied. Therefore, it is very important to select suitable microorganisms and study the effects of externally added microbes on the structure and function of microbial communities.

Cigars are one of the oldest traditional tobacco fermented products (Reid et al., 1937). Flavor-producing microbes play an important role in tobacco fermentation (Liu et al., 2021). In our previous work, we found that *Acinetobacter* were the important producers of the aldehydes and ketones. And we isolated two flavor producers, *Acinetobacter* sp. 1H8 and *Acinetobacter indicus* 3B2 from high-quality cigar tobacco leaves (CTLs). We speculated that their addition could improve the quality of some ordinary CTLs. In this study, we investigated whether these two extrinsic stains could improve the quality and flavor of the CTLs and their effects on the structure and function of bacterial communities.

## Materials and Methods

### Strains

The strains used in this study were isolated from the CTLs and deposited in the China General Microbiological Culture Collection Center (CGMCC): *Acinetobacter* sp. 1H8 CGMCC NO.23678 and *Acinetobacter indicus* 3B2 CGMCC NO.23679.

### Inoculation Experiment

The fermentation medium for culturing strains was prepared with sucrose 20 g/l, peptone 20 g/l, yeast powder 15 g/l, K_2_HPO_4_ 1.5 g/l NaH_2_PO_4_ 3 g/l, and autoclaved at 121°C for 15 min. Strains were inoculated by sterile loops in 250-ml flasks with 50 ml fermentation medium, and cultured at 220 rpm 30°C for 36 h. Then the fermentation broth was inoculated into CTLs with 30% inoculation amount (v/g) and fermented in a biochemical incubator. All fermentations were conducted without agitation at 30°C for 15 days. The unfermented CTLs and uninoculated CTLs were prepared as Control 1 and Control 2, respectively. All experiments were performed in triplicate. The quality of CTLs was blindly assessed by three professional tasters. With 10–20 years of testing experience, these tobacco tasters have conducted sensory evaluation on more than 2,000 cigar samples, and can accurately, consistently, and repeatedly evaluate cigars.

### Volatile Flavor Compound Analysis

Volatile flavor compounds (VFCs) in CTLs were analyzed by headspace solid phase microextraction-gas chromatography–mass spectrometry (HS-SPME-GC–MS). CTLs were dried at 40°C and pulverized by a grinder. 1.5-g powder was placed in a 10 ml glass vial and extracted by headspace solid-phase microextraction (50/30 μm DVB/CAR/PDMS fibre, Supelco, Bellefonte, PA, United States) at 60°C for 30 min. After extraction, volatile flavor compounds (VCFs) were identified using a Pegasus BT GC-TOFMS (LECO Co., St. Joseph, MI, United States), with a DB-5MS column (60 m × 0.25 mm id × 0.25 μm film thickness). Helium C-60 was used as a carrier gas with a flow rate of 1 ml/min, and the injector port was heated to 250°C. The oven temperature was fixed at 40°C for 2 min, increased to 250°C at a rate 10°C/min, and then held for 5 min. Meanwhile, the transfer line and ion source temperatures were maintained at 280°C and 210°C, respectively. Electron impact (EI) was used as the ionization mode, with an EI voltage of 70 eV, and a mass scan range of 33–400 m/z was used for full-scan mode with an acquisition rate of 10 scans/s.

The acquired GC–MS raw data from the QC sample was analyzed with the Automatic Mass Spectral Deconvolution and Identification System (AMDIS) to verify individual analytes’ presence and deconvolute the co-eluting peaks as previously described. The parameters for the deconvolution were set as follows: component width = 20, adjacent peaks subtraction = 2, resolution = high, sensitivity = low, and shape requirements = medium. The parameters for peak detection were set at the default values. The detected compounds with an abundance of <1,000 and a signal-to-noise value of <50 were removed to discard identification errors or duplication. Compounds were identified by comparing the mass spectra and retention index with those in the NIST08 Mass Spectral Database, the Agilent Fiehn Metabolomics Retention Time Locked (RTL) Library, and an in-house library focused on tobacco metabolites. Standards for constructing the in-house library were prepared as described above. A SCAN + SIM method and an in-house automatic integration method were then established in the Agilent MSD Chem Station and applied to quantify the selective ion traces as previously described. Manual corrections were performed to guarantee the accuracy of the integration. A three-dimensional matrix was generated, including the sample information, peak retention time, and relative peak intensities. Internal standards and any known artificial peaks, such as peaks caused by noise, column bleed, and derivatization procedures, were removed from the matrix.

### Bacterial Community Analysis

To collect microbes from the CTLs, ~5-g CTLs were added to a 250-ml flask containing 100-ml filtered normal saline (0.9% NaCl, pH 7) and oscillated at 4°C, 220 rpm for 4 h. CTLs were removed by gauze, and microbes were collected by centrifuging at 7,000 × *g* for 15 min. Total microbial genomic DNA was extracted using the DNeasy PowerSoil Kit (QIAGEN, Inc., Netherlands), following the manufacturer’s instructions, and stored at −20°C until further analysis. The quality of extracted DNA was measured by agarose gel electrophoresis. PCR amplification of the bacterial and archaeal 16S rRNA genes V4–V5 region was performed using the forward primer 515F (Parada et al., 2016; 5′-GTGCCAGCMGCCGCGGTAA-3′) and the reverse primer 907R (Morales and Holben, 2009; 5′-CCGTCAATTCMTTTRAGTTT-3′). Sample-specific 7-bp barcodes were incorporated into the primers for multiplex sequencing. The final PCR volume was 25 μl and consisted of 5 μl of Q5 reaction buffer (5×), 5 μl of Q5 High-Fidelity GC buffer (5×), 0.25 μl of Q5 High-Fidelity DNA Polymerase (5 U/μl), 2 μl (2.5 mM) of dNTPs, 1 μl 515F primer (10 μM; final: 0.4 μM), 1 μl 806R primer (10 μM, final: 0.4 μM), 2 μl of DNA Template, and 8.75 μl nuclease-free water. The thermocycler conditions were: initial denaturation at 98°C for 2 min followed by 28 cycles consisting of denaturation at 98°C for 15 s, annealing at 55°C for 30 s, and extension at 72°C for 30 s, with a final extension of 5 min at 72°C. PCR products were purified with Agencourt AMPure Beads (Beckman Coulter, IN) and quantified using the PicoGreen dsDNA Assay Kit (Invitrogen, Carlsbad, CA, United States). After the individual quantification step, amplicons were pooled in equal amounts, and paired-end 2 × 300 bp sequencing was performed using the Illumina MiSeq platform with MiSeq Reagent Kit v3.

The 16S rRNA gene sequences were processed using QIIME 2 (Bolyen et al., 2019). Briefly, raw sequencing reads with exact matches to the barcodes were assigned to respective samples and identified as valid sequences. The low-quality sequences were filtered through the following criteria (Chen and Jiang, 2014): sequences that had a length of <150 bp, sequences that had average Phred scores of <20, sequences that contained ambiguous bases, and sequences that contained mononucleotide repeats of >8 bp. Paired-end reads were assembled using FLASH (Magoc and Salzberg, 2011). After chimera detection, the remaining high-quality sequences were clustered into amplicon sequence variants (ASVs). Taxonomic classification was performed using the plugin q2-feature-classifier using the classify-sklearn method (Pedregosa et al., 2011) and the pre-trained SILVA version 132 database (Quast et al., 2013) with 99% similarity. Alpha diversity indexes were calculated by using the command “qiime diversity alpha-rarefaction.” PICRUSt (Phylogenetic Investigation of Communities by Reconstruction of Unobserved States) function prediction was performed using bacterial 16S rRNA sequencing data to determine the abundance of microbial functional genes in the KEGG metabolic pathway (Langille et al., 2013).

### Statistical Analysis

Heat maps and cluster analyses were performed in the statistical environment R v. 4.0.0. The Galaxy^1^ was used for linear discriminant analysis effect size (LEfSe) analysis to assess significant differences of CTLs with different treatments. Additionally, the correlation between the representative bacteria and core VFCs based on Spearman’s correlation coefficients (*p* < 0.05, |r| > 0.3), network analysis was performed by using Gephi software.

## Results

### Bacterial Composition Varied After Inoculation and Fermentation

**Figure 1:**
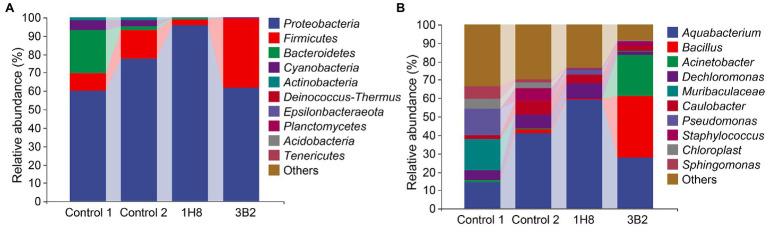
Bacterial communities in cigar tobacco leaves. The top 10 predominant bacterial phyla **(A)** and genera **(B)**.

To explore the influence of the inoculation of *Acinetobacter* on original bacterial community, bacteria in CTLs with different treatments were sequenced. The high-throughput sequencing generated 642,151 sequence reads from sequence reads from 12 CTLs. After the quality control processing, including filtered, denoised, merged, and nonchimeric, there were 483,897 high-quality sequences, with an average of 40,324 sequences, and obtained 1,914 ASVs. The abundance of bacterial taxa was shown in Figure 1, *Proteobacteria*, *Firmicutes*, and *Bacteroidetes* were predominant phyla, *Aquabacterium*, *Bacillus*, *Acinetobacter*, *Muribaculaceae*, and *Pseudomonas* were predominant genera. There was no doubt that fermentation changed the composition of bacterial communities. At phyla level, the abundance of *Proteobacteria* and *Firmicutes* increased from 59.9%5 and 9.98% to 77.59% and 15.58% after fermentation, respectively, while the abundance of *Bacteroidetes* decreased from 23.10% to 2.16%. At genera level, the abundance of *Aquabacterium* increased from 14.26% to 41.08%, while the abundance of *Muribaculaceae* and *Pseudomonas* decreased from 17% and 14.04% to <0.01%. Similarly, external microorganisms also changed the composition of bacterial communities. At phyla level, the abundance of *Proteobacteria* increased from 59.95% to 95.61% after inoculation with *Acinetobacter* sp. 1H8 and decreased to 37.63% after inoculation with *Acinetobacter indicus* 3B2, while the abundance of *Bacteroidetes* decreased from 15.58% to 3.1% after inoculation with *Acinetobacter* sp. 1H8 and increased to 37.63% after inoculation with *Acinetobacter indicus* 3B2. At genera level, the abundance of *Aquabacterium* increased from 41.08% to 59.01% after inoculation with *Acinetobacter* sp. 1H8 and decreased to 27.61% after inoculation with *Acinetobacter indicus* 3B2. The abundance of *Bacillus* and *Acinetobacter* increased from 1.83% and 0.62% to 33.68% and 22.43% after inoculation with *Acinetobacter indicus* 3B2, respectively.

**Figure 2:**
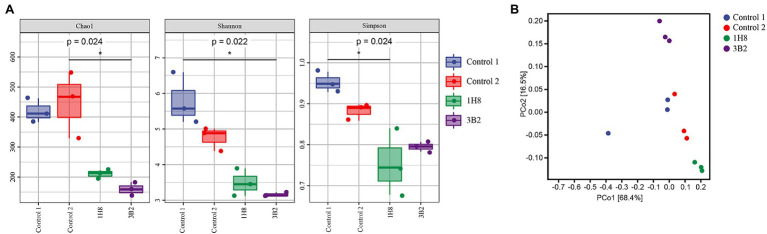
Diversity of bacterial communities in cigar tobacco leaves. Alpha diversity was determined based on the Chao1 index, the Shannon index, and the Simpson index **(A)**, Beta diversity was measured by weighted UniFrac distance **(B)**.

The alpha diversity of the bacterial community was evaluated by indices including Chao1 index, Shannon index, and Simpson index, in which the first and the latter two represent richness and diversity, respectively (Figure 2A). They all showed external microorganisms significantly reduced the richness and evenness of original bacterial community. Unconstrained principal coordinate analysis (PCoA) of Weighted-unifrac distance revealed that the microbiota of CTLs with different treatment formed three distinct clusters (Figure 2B), which separated along the second coordinate axis. As expected, the inoculation of *Acinetobacter* indisputably altered the structure of bacterial community.

### Significant Different Microbes After Inoculation and Fermentation

**Figure 3:**
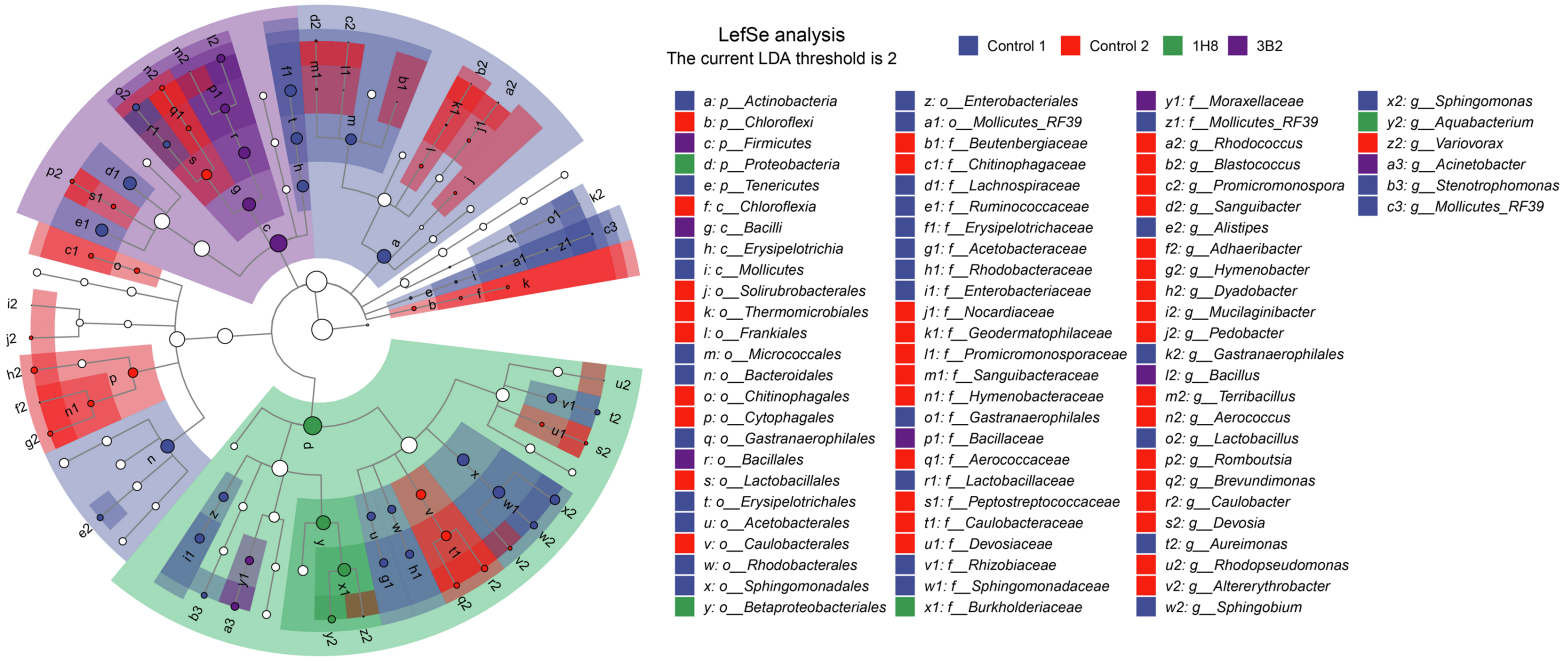
Evolutionary branch map of the bacteria with significantly different in cigar tobacco leaves with different treatments.

To explore the different bacteria among CTLs with different treatments, LEfSe analysis was conducted to reveal the significant differences below the level of phylum (Figure 3). The circles from inner to outer represents bacteria classification from phylum to genus levels, and corresponding colors in every group denotes bacteria taxa with a significant difference. Notably, 81 different bacteria appeared in the LDA threshold of 2 judging by statistically significant differences (*p* < 0.05), which consist of 5 phyla, 4 classes, 18 orders, 25 families, and 29 genera. In detail, 17 genera were significantly enriched in uninoculated CTLs, such as *Rhodococcus*, *Blastococcus*, *Promicromonospora*, and *Sanguibacter*. Eight genera were significantly enriched in unfermented CTLs, such as *Lactobacillus*, *Sphingobium*, and *Rhizopus*. Two genera were significantly enriched in CTLs inoculated *Acinetobacter indicus* 3B2, such as *Bacillus* and *Acinetobacter*. Only *Aquabacterium* was significantly enriched in CTLs inoculated *Acinetobacter* sp. 1H8.

### Changes in Bacterial Metabolic Pathways After Inoculation and Fermentation

**Figure 4:**
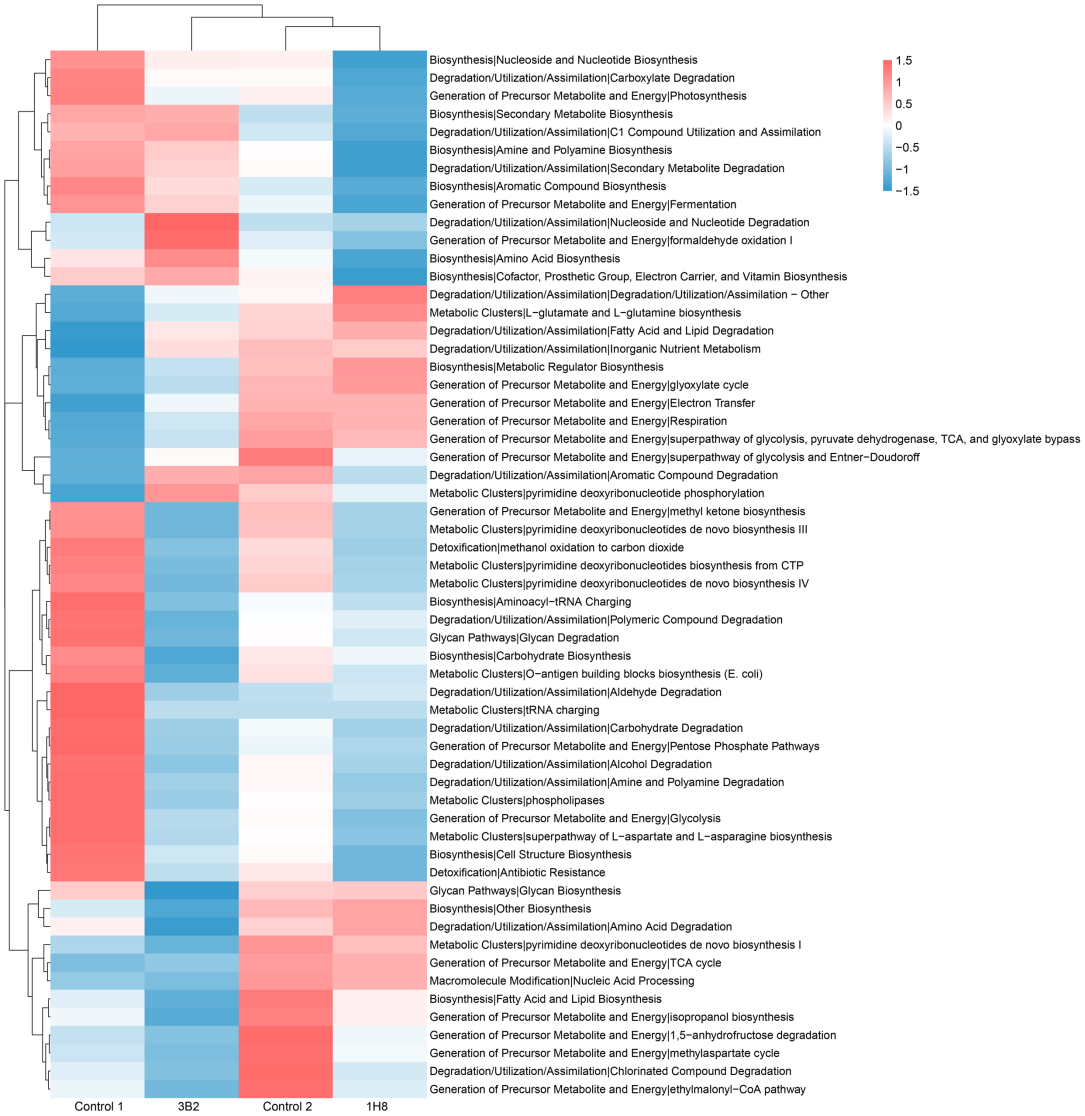
The relative abundance of bacterial metabolic pathway in cigar tobacco leaves.

**Figure 5:**
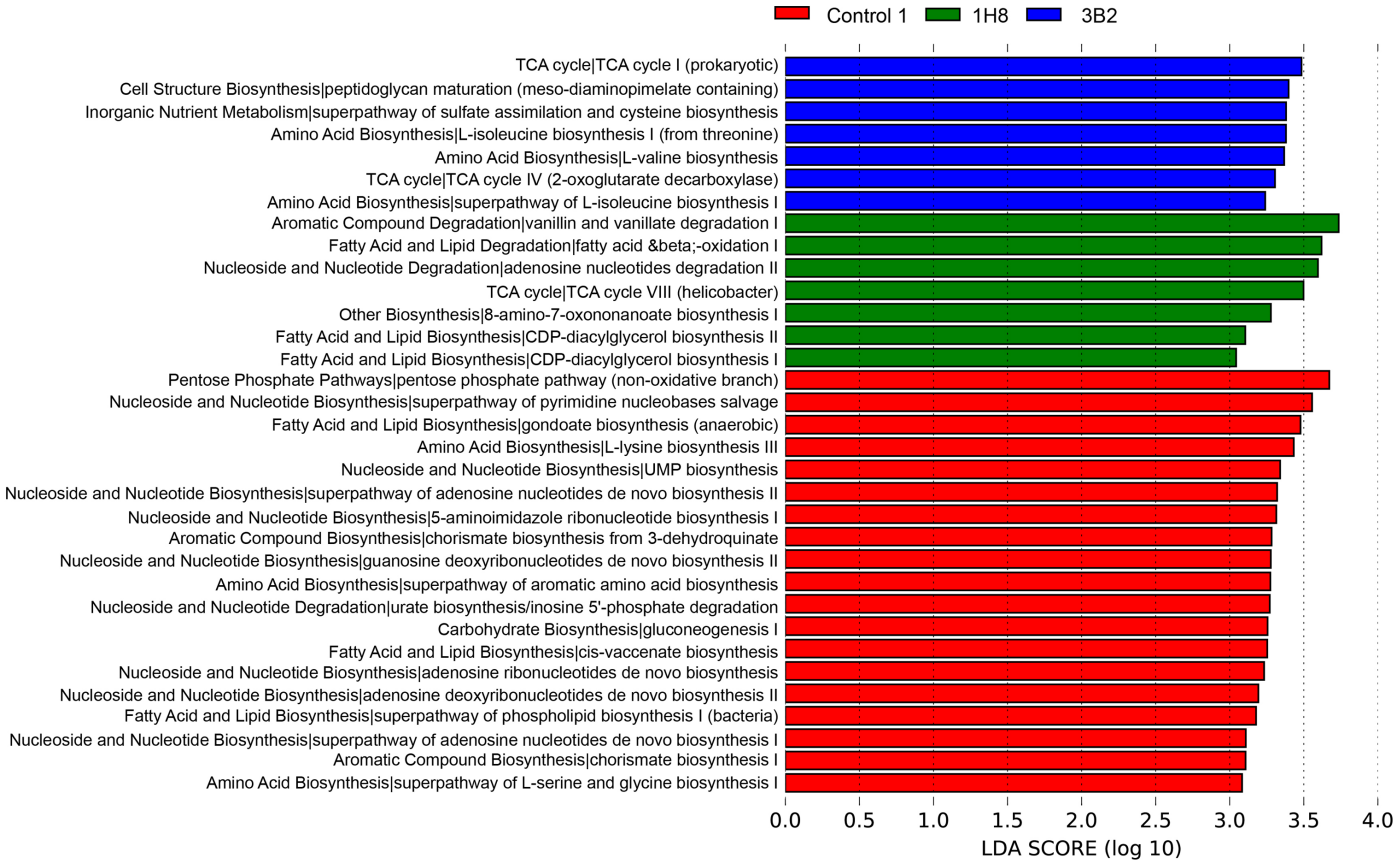
Significantly different bacterial metabolic pathway in cigar tobacco leaves with different treatments.

PICRUSt analysis results showed that there were more functional genes annotated to biosynthesis, degradation/utilization/assimilation, generation of precursor metabolite and energy, glycan pathways, macromolecule modification, and metabolic clusters (Figure 4). The cluster analysis identified the CTLs inoculated *Acinetobacter* sp. IH8 and uninoculated CTLs were classified into one cluster with the similar abundance of bacterial metabolic pathway, this may be because they have similar bacterial community. The addition of external microorganisms not only changed the structure of bacterial community, but also changed the function of bacterial community. The abundance of a large number of bacterial metabolic pathways decreased significantly after inoculation. LEfSe analysis was conducted to identify the significant different bacterial metabolic pathways in CTLs with different treatments (Figure 5). Metabolic pathways involved in biosynthesis, including fatty acid and lipid biosynthesis, amino acid biosynthesis, nucleoside and nucleotide biosynthesis, aromatic compound biosynthesis, and carbohydrate biosynthesis, were significantly enriched in unfermented CTLs. Metabolic pathways involved in degradation, including aromatic compound degradation, fatty acid and lipid degradation, nucleoside and nucleotide degradation, were significantly enriched in CTLs inoculated *Acinetobacter* sp. IH8. Metabolic pathways involved in cell growth, including cell structure biosynthesis, inorganic nutrient metabolism, amino acid biosynthesis, and TCA cycle, were significantly enriched in CTLs inoculated *Acinetobacter indicus* 3B2.

### Interactions Between Extrinsic and the Intrinsic Microbes

**Figure 6:**
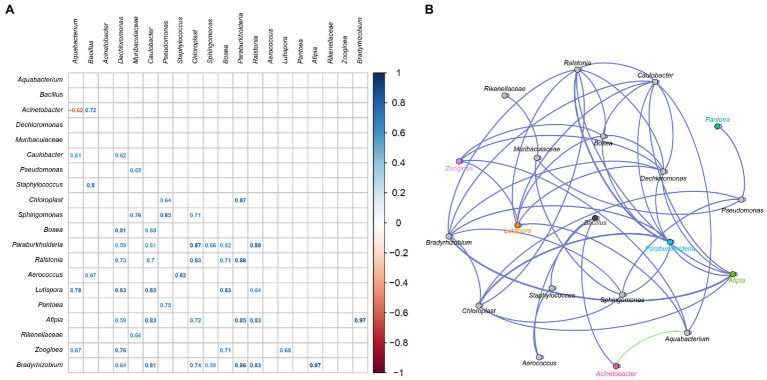
The correlation between bacterial community. Heatmap analysis based on Spearman’s correlation coefficients between representative bacterial taxa **(A)**. Co-occurrence networks of representative bacterial taxa. Green line means positive correlation; red line means negative correlation **(B)**.

Microbial interactions are the main factors shaping community structure (Mould and Hogan, 2021). To elucidate interactions between the inoculated strains and the native bacteria, an association network was established based on bacterial abundance (Kumar et al., 2019). As showed in Figure 6, *Acinetobacter* was negative correlative with *Aquabacterium*, and was positive correlative with *Bacillus*. There must be a non-cooperative relationship between *Acinetobacter* and *Aquabacterium*, such as competition, parasitism, predation, and antagonism. There must also be a cooperative relationship of mutualism and symbiosis between *Acinetobacter* and *Bacillus*. The inoculated *Acinetobacter* 1H8 was inhibited by *Aquabacterium*, while inoculated *Acinetobacter indicus* 3B2 inhibited *Aquabacterium* and promoted *Bacillus*. They in turn affected other microbiotas, such as *Staphylococcus*, *Aerococcus*, *Lutispora*, and *Zoogloea*. In addition to the negative correlation between *Acinetobacter* and *Aquabacterium*, other interactions were positive.

### Changes in the Profiles of Volatile Flavor Compounds After Inoculation and Fermentation

**Figure 7:**
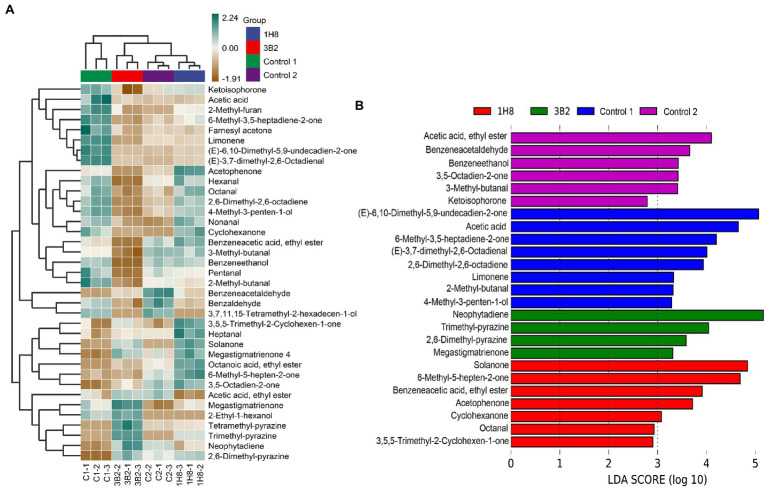
Volatile flavor compounds in the cigar tobacco leaves. Hierarchical clustering of volatile flavor compounds in the cigar tobacco leaves **(A)**, Different volatile flavor compounds in different cigar tobacco leaves **(B)**.

*Acinetobacter* were found to be the main producer of produce aldehydes and ketones in CTLs in our previous studies. They could produce flavor-related aldehydes and ketones in a simple synthetic medium, such as benzaldehyde, phenylacetaldehyde, 4-hydroxy-3-ethoxy-benzaldehyde, and 3,5,5-trimethyl-2-cyclohexene-1-one. There is no doubt that they are able to produce more products in CTLs because they have more nutrients available. To investigate the effect of extrinsic strains on the metabolic activity of the original microbial community, the VFCs in CTLs were determined. In total, 37 VFCs were selected among hundreds of compounds based on content and co-detection for further analysis, which included 4 alcohols, 9 aldehydes, 13 ketones, 3 esters, 3 pyrazines, 3 alkene,1 furan, and 1 acid. As shown in Figure 7A, the cluster analysis divided unfermented and fermented CTLs into two clusters, the vast majority of VFCs have changed after fermentation. For example, benzeneacetaldehyde, benzaldehyde, and 3,7,11,15-tetramethyl-2-hexadecen-1-ol were obviously increased in uninoculated CTLs after fermentation. Megastigmatrienone, 2-ethyl-1-hexanol, tetramethyl-pyrazine, trimethyl-pyrazine, neophytadiene, pyrazine, and 2,6-dimethyl were obviously increased in CTLs inoculated *Acinetobacter indicus* 3B2. 3,5,5-Trimethyl-2-cyclohexen-1-one, heptanal, solanone, megastigmatrienone 4, octanoic acid, ethyl ester, 3,5-octadien-2-one, and 6-methyl-5-hepten-2-one were obviously increased in CTLs inoculated *Acinetobacter* sp. 1H8. While ketoisophorone, acetic acid, 2-methyl-furan, 6-methyl-3,5-heptadiene-2-one, farnesyl acetone, limonene, (E)-6,10-dimethyl-5,9-undecadien-2-one, and (E)-3,7-dimethyl-2,6-octadienal were decreased after fermentation. VFCs of inoculated CTLs varied significantly compared to uninoculated CTLs.

LEfSe analysis was used to identify the significant different VFCs in CTLs with different treatments (Figure 7B). Among the 37 VFCs, 26 VFCs appeared in the LDA threshold of 2 judging by statistically significant differences (*p* < 0.05). In detail, six VFCs were significantly enriched in uninoculated CTLs, such as acetic acid, ethyl ester, benzeneacetaldehyde, and benzeneethanol. Eight VFCs were significantly enriched in unfermented CTLs, such as (E)-6,10-dimethyl-5,9-undecadien-2-one, acetic acid, and 6-methyl-3,5-heptadiene-2-one. Four VFCs were significantly enriched in CTLs inoculated *Acinetobacter indicus* 3B2, including neophytadiene, tetramethyl-pyrazine, 2,6-dimethyl-pyrazine, and megastigmatrienone. Seven VFCs were significantly enriched in CTLs inoculated *Acinetobacter* sp. 1H8, such as solanone, 6-methyl-5-hepten-2-one, and benzeneacetic acid, ethyl ester.

### Correlation Analysis of the Predominant Bacteria and VFCs

**Figure 8:**
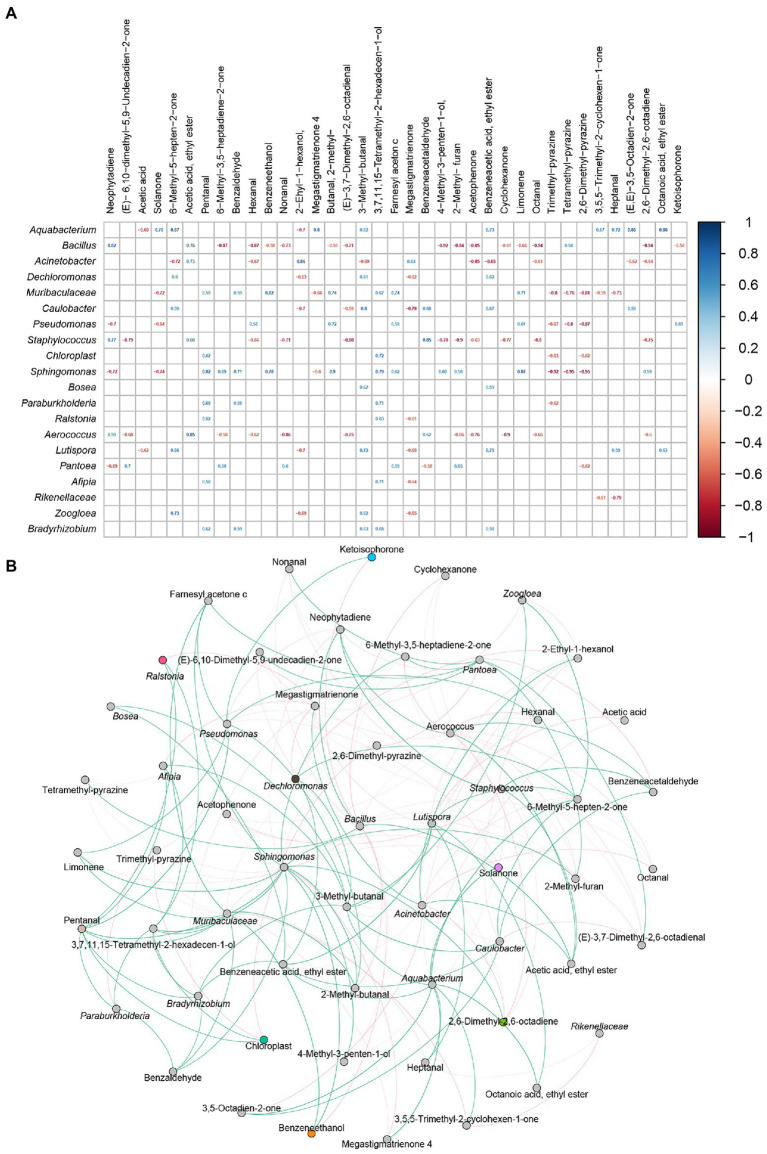
The correlation between bacterial community and volatile flavor compounds. Heatmap analysis based on Spearman’s correlation coefficients between representative bacteria and core volatile flavor compounds **(A)**. Co-occurrence networks of representative bacterial taxa and core volatile flavor compounds. Green line means positive correlation; red line means negative correlation **(B)**.

The changes of volatile flavor compounds were mainly caused by microbial metabolism, so it was very important to determine the relationship between bacteria and VFCs. The relationships between VFCs (*n*:37) and representative bacteria (*n*: 20) were analyzed by Spearman’s correlation coefficients and visualized by Gephi. As shown in Figure 8, *Acinetobacter* were positively related to acetic acid, ethyl ester, 2-ethyl-1-hexanol, and megastigmatrienone, and negatively related to 6-methyl-5-hepten-2-one, hexanal, 3-methyl−butanal, acetophenone, benzeneacetic acid, ethyl ester, octanal, 3,5-octadien-2-one, and 2,6-dimethyl-2,6-octadiene. *Aquabacterium* and *bacillus*, which were significantly associated with *Acinetobacter*, were also related to many volatile flavor compounds. *Aquabacterium* was positively related to most volatile flavor compounds, while *Bacillus* was negatively related to most volatile flavor compounds.

### Changes in Flavor of Cigar Tobacco Leaves After Inoculation and Fermentation

Not surprising, the quality of CTLs was significantly changed after inoculation and fermentation. Surprisingly, however, the tasters gave the same evaluation to CTLs inoculated different strains. They felt the bean fragrance, mellow, smooth, aftertaste, sweetness, cleanliness, and aftertaste were significantly improved, and the impurities and irritation were reduced. By analyzing the function of VFCs, it was found that solanone may be the main force to improve the quality of CTLs, because it could reduce impurities and irritation, and increase the mellow, fluency, lingering, sweetness, cleanliness, and aftertaste of CTLs. Bean flavor might be composed of different combinations and proportions of VFCs.

## Discussion

This work revealed that the quality of CTLs were improved by the addition of extrinsic microbes. Changes in the quality of CTLs were not only related to the flavor production ability of the extrinsic microbes, but also to the interaction between the external and internal microbes. Microbial interactions were important forces in reconstructing the microbial community (Romdhane et al., 2022). The interactions between the external and internal microbes changed the original microbial community. Meanwhile, changes in microbial community led to variations in VFCs and quality of CTLs. Therefore, it is important to gain deep insight into these changes and the underlying reasons for these changes.

In this work, two extrinsic microbes (*Acinetobacte*r sp. 1H8 and *Acinetobacter* indicus 3B2) were inoculated into CTLs, they exerted different effects on the original microbial community. The abundance of *Acinetobacter* remained stable in uninoculated CTLs after fermentation. When *Acinetobacte*r sp. 1H8 was inoculated in CTLs, this strain was completely inhibited by the native microbiotas, the abundance of *Acinetobacter* was significantly reduced after fermentation. The inoculation of *Acinetobacte*r sp. 1H8 caused the endogenous microbes to turn from competition to cooperation to compete together against the extrinsic microbes to suppress the extrinsic perturbation. Normally, the microbial community remains stable due to the complete suppression of extrinsic microbes by endogenous microbes (Wu et al., 2016). However, although *Acinetobacte*r sp. 1H8 grew poorly, it still influenced the overall metabolic activity of the intrinsic microbes. *Aquabacterium* proliferated wildly upon stimulation by exogenous microorganisms, their abundance was significantly increased. Its influence has had a positive effect. From the perspective of the overall change of microbial community, the inoculation of *Acinetobacter* sp. 1H8 enhanced the normal evolutionary trend of bacterial community. Similar result was also found in in the cocultures of *Metschnikowia pulcherrima* and *S. cerevisiae*, the positive effect leads to increases in the types and amounts of metabolites, such as fatty acids, ethyl esters and acetates, and terpinol (Sadoudi et al., 2012). It may be helpful to promote the succession of microbial community, accelerated the fermentation process, and shortened the fermentation time (Zhang et al., 2021). When *Acinetobacter indicus 3B2* was inoculated in CTLs, they were successfully colonized CTLs, the abundance of *Acinetobacter* was significantly increased after fermentation, meanwhile, they also caused an increase in the abundance of *Bacillus* and a decrease in the abundance of *aquabacterium.* The inoculation of *Acinetobacter indicus* 3B2 greatly changed structure of the microbial community in CTLs. *Acinetobacter indicus 3B2* might be considered keystone specie in CTLs, which has an extremely high impact on a particular ecosystem (Rottjers and Faust, 2019). It was also critical for the overall structure and function of an ecosystem (Banerjee et al., 2018). The inoculation of *Acinetobacte*r also significantly changed the metabolic functions in bacterial communities. Metabolic pathways involved in degradation were significantly enriched in CTLs inoculated *Acinetobacter* sp. IH8. The inoculation of *Acinetobacter* sp. IH8 significantly increased the degradation ability of macromolecular substances in microbial community, it would increase precursors of VFCs. The inoculation of *Acinetobacter indicus* 3B2 significantly promoted the growth of some functional microbiotas, such as *bacillus* and *Acinetobacter*, which were found to be the main producers of aldehyde and ketones in CTLs in our previous study. Microbial interaction analysis found *Acinetobacter* was negative correlative with *Aquabacterium*, and was positive correlative with *Bacillus*.

*Acinetobacter* were found to be the main producer of produce aldehydes and ketones in CTLs in our previous studies. They could produce flavor-related aldehydes and ketones in a simple synthetic medium, such as benzaldehyde, phenylacetaldehyde, 4-hydroxy-3-ethoxy-benzaldehyde, and 3,5,5-trimethyl-2-cyclohexene-1-one. There is no doubt that they are able to produce more products in CTLs because they have more nutrients available. Their inoculation also greatly changed volatile flavor compound profile of CTLs. The inoculation of *Acinetobacter* sp. 1H8 significantly increased the content of solanone, 6-methyl-5-hepten-2-one, benzeneacetic acid, ethyl ester, cyclohexanone, octanal, acetophenone, and 3,5,5-trimethyl-2-cyclohexen-1-one. The inoculation of *Acinetobacter indicus* 3B2 significantly increased the content of trimethyl-pyrazine, 2,6-dimethyl-pyrazine, and megastigmatrienone. These VFCs have an important contribution to the flavor of CTLs. For example, solanone may be the main force to reduce impurities and irritation, and increase the mellow, fluency, lingering, sweetness, cleanliness, and aftertaste of CTLs. Its content was greatly increased in CTLs inoculated both microorganisms. The increase of pyrazine would enhance baked, roasted, rosy, and honey-like aroma. However, due to the increase and decrease of a variety of VFCs, the mixed flavor compounds produce a new flavor. Bean flavor might be composed of a variety of VFCs, and there were differences in the composition of the two CTLs. This may be the main reason why CTLs inoculated different microorganisms showed a flavor characteristic.

## Conclusion

When some traditional fermented products that are produced by spontaneous fermentation unable to meet the demands of consumers, inoculating extrinsic microbes may improve these products. In this work, we demonstrate that the inoculation of two extrinsic microbes (*Acinetobacter* sp. 1H8 and *Acinetobacter indicus* 3B2) improved the quality and flavor of CTLs. Inoculated microbes can not only exert their own metabolic ability, but also affect the structure and function of native microbial community. We revealed that the interaction between exogenous microorganisms and native microbes, the different effects of exogenous microorganisms on original microbial community, the association between microbes and VFCs, and the formation mechanism of tobacco flavor. Collectively, our present work has proved the effect of inoculated microorganisms in traditional fermentation industry and elucidated changes in the overall structure and function of microbial communities after inoculation. These results suggest that controlling the microbial community could significantly improve the quality and safety of fermentation products, and this work may provide a good way to gain insight into the microbial ecosystem of traditional fermentation.

## Data Availability Statement

The datasets presented in this study can be found in online repositories. The names of the repository/repositories and accession number(s) can be found at: NCBI SRA BioProject, accession no: PRJNA813020.

## Author Contributions

TZ: conceptualization, data curation, formal analysis, methodology, software, and writing—original draft. QiZ, QW, and PL: investigation, methodology, and resources. XW, QuZ, and WC: methodology, resources, and project administration. JZ, GD, and DL: funding acquisition, supervision, and writing—review and editing. All authors contributed to the article and approved the submitted version.

## Funding

This work was supported by the National Key Research and Development Program of China (2019YFC1605800), the China National Tobacco Corporation 2020 Major Science and Technology Project 110202001040(XJ-02), and the Major projects on constructing the “mellow and sweet fragrance styles” of Chinese-Stylistic Tobacco (ctx201905).

## Conflict of Interest

QiZ, DL, PL, QuZ, and WC were employed by China Tobacco Sichuan Industrial Co., Ltd.

The remaining authors declare that the research was conducted in the absence of any commercial or financial relationships that could be construed as a potential conflict of interest.

## Publisher’s Note

All claims expressed in this article are solely those of the authors and do not necessarily represent those of their affiliated organizations, or those of the publisher, the editors and the reviewers. Any product that may be evaluated in this article, or claim that may be made by its manufacturer, is not guaranteed or endorsed by the publisher.

## Footnotes

^1^ https://huttenhower.sph.harvard.edu/galaxy/
